# Mitochondria-Associated Membrane Scaffolding with Endoplasmic Reticulum: A Dynamic Pathway of Developmental Disease

**DOI:** 10.3389/fmolb.2022.908721

**Published:** 2022-06-14

**Authors:** Russell P. Saneto, Francisco A. Perez

**Affiliations:** ^1^ Division of Pediatric Neurology, Department of Neurology, Seattle Children’s Hospital/University of Washington, Seattle, WA, United States; ^2^ Neuroscience Institute, Center for Integrated Brain Research, Seattle Children’s Hospital, Seattle, WA, United States; ^3^ Department of Radiology, Seattle Children’s Hospital/University of Washington, Seattle, WA, United States

**Keywords:** mitochondria-associated endoplasmic reticulum membrane, calcium, autophagy, phospholipids, fatty acid metabolism, gene products, metabolism

## Abstract

Communication between intracellular organelles is essential for overall cellular function. How this communication occurs and under what circumstances alterations transpire are only the beginning to be elucidated. The pathways of calcium homeostasis, lipid transfer, mitochondrial dynamics, and mitophagy/apoptosis have been linked to the endoplasmic reticulum and tethering sites on the outer and/or inner mitochondrial membrane called mitochondria-associated endoplasmic reticulum membranes (MAM). Sensitive visualization by high-powered microscopy coupled with the advent of massive parallel sequencing has elaborated the structure, while patient’s diseases have uncovered the physiological function of these networks. Using specific patient examples from our pediatric mitochondrial center, we expand how specific genetic pathological variants in certain MAM structures induce disease. Genetic variants in *MICU1*, *PASC-2*, *CYP2U1*, *SERAC1*, and *TANGO2* can induce early development abnormalities in the areas of cognition, motor, and central nervous system structures across multiple MAM pathways and implicate mitochondrial dysregulation.

## Introduction

Historically, cellular research has concentrated on understanding how individual intracellular components encode cellular specificity. Intracellular organelles perform specific and unique biochemical reactions that dictate multiple aspects of cellular physiology. These organelle’s metabolic functions are isolated by membrane or lipid scaffolding that create the compartmentalization necessary for specific protein, lipid, hormonal, and carbohydrate synthesis to occur. However, specialized organelle metabolism also requires a mechanism to communicate between compartments for cell functioning. There is growing evidence that specific organelle contact sites may be important in this communication. Early microscopic structural studies identified close proximation and specific tether proteins connecting the endoplasmic reticulum (ER) membrane and mitochondria, which occur under certain physiological stressors such as fasting and refeeding or regeneration (Bernhard and Rouiller, 1956; Shore and Tata, 1977; Meier et al., 1981; Csordás et al., 2006). These subcellular enriched sites have been named mitochondria-associated endoplasmic reticulum membranes (MAM; Vance, 1990). Specificity of their make-up, location on the membrane, and possible physiological functions became evident through gene discovery linking MAM structural proteins with identified disease phenotypes.

Using yeast models, specific mitochondrial and ER contact sites (MERCS) have been documented by confocal and lattice light sheet instrumentation (Chen et al., 2014; Valm et al., 2017). Valm et al. found that mitochondria interact predominantly with the ER but also with Golgi apparatus and lipid droplets (Valm et al., 2017). These specialized contact sites promote the exchange of molecules and proteins with associated proteins and lipids that stabilize sites (Pribasnig et al., 2018). The full proteome of MERCS is under intense study and resemble mammalian MAMs, but the complete spectrum remains unknown. Currently, there are over 100 proteins associated with a structural or biochemical role in these contact sites. The ultrastructure and composition of MERCS/MAM is beyond the scope of this study but has been reviewed in several excellent studies (Schon and Area-Gomez, 2013; Pailluson et al., 2016; Prinz et al., 2020).

### Mitochondria

Structurally, a mitochondrion is composed of two double-layered lipid membranes: an outer mitochondrial membrane (OMM) and an inner mitochondrial membrane (IMM) separated by an intermembrane space (IMS). Within the IMM lies the matrix where multiple physiological processes take place (reviewed in Frazier et al., 2019; Saneto, 2020). Each membrane structure has a unique set of integrated translocase proteins to transport substrates across the outer mitochondrial membrane (TOMs) and inner mitochondrial membrane (TIMMs) into the matrix. Within the matrix, these proteins/lipids are combined with the matrix mitochondrial products for mitochondrial functioning.

### Endoplasmic Reticulum

The nucleus is connected to the cytosol by the ER. The ER is a continuous phospholipid bilayer that is composed of tubules or flattened sacs. Within the bilayer are specific proteins and chaperones. Proteins are synthetized in the lipid bilayer by a specialized network of mRNA that allows translation of proteins to reduce exposure of hydrophobic regions and misfolding. This specialized network gives a rough appearance on transmission electron microscopy called the “rough ER.” The rough ER is responsible for specific protein transport into the mitochondria. At least 20 proteins have been associated specifically to the rough ER (Alberts et al., 2015). The specialized ER that does not contain ribosomes is termed “smooth ER” where vesicles transport and manufacture a group of synthesized proteins, hormones, and lipids to distribute to the cell. It is also responsible for the detoxification of water-insoluble drugs and toxins as well as calcium storage (Alberts et al., 2015).

Current literature indicates that the mitochondrial-ER tethering has physiological functioning essential for several cellular pathways. Specific roles in calcium homeostasis (Rizzuto et al., 1998; Wu et al., 2018), lipid transfer (Lev, 2012), mitochondrial dynamics (Hoppins and Nunnari, 2012), and mitophagy/apoptosis (Youle and Narendra, 2011; Youle and van der Bliek, 2012) have been described.

Here, we present selected medical case studies in children with genetic variants in MAM-related pathway proteins to highlight the importance of these biochemical processes and the range of disease phenotypes. Specifically, we describe human diseases related to dysfunction of calcium shuttling, autophagosome formation, phospholipid remodeling, fatty acid metabolism, and mixed Golgi and mitochondria–ER transport (Table 1). Our series highlights the challenges of separating primary and secondary mitochondrial dysfunction; nevertheless, these pathways are necessary for proper mitochondrial function and are clearly altered by changes in specific proteins within individual MAMs. These patients also demonstrate distinct neuroimaging features which may guide diagnostic recognition.

**TABLE 1:** Gene variants and disease-related proteins involved in ER-mitochondrial coupling, MAMs Gene Variants Function Protein and Location in MAM.

Gene variants	Function	Protein and location in MAM
*MICU1*(c.161+1G>A/c.386G>C)	Calcium shuttling: regulation of calcium	MICU1: IMS
*PACS-2*(c.625G>A)	Regulation of ER-mitochondria	PACS-2: Interspace
Communication: Autophagosome Formation between OMM and ER		
*CYP2U1*(c.342C>A/c.1001del A	Fatty acid metabolism enzymes: PS and PE	CYP2U1: IMM
Decarboxylation		
*SERAC1+*(c.1763del T/c.1763del T)	Phospholipid remodeling	SERAC1:ER Membrane
*TANGO2* (del exon 3–9/c.711–3C>G	Unknown in humans	TANGO2: Golgi ER
(del exon 3–9/del 3–9)		Membrane-OMM

*MICU1*: *mitochondrial calcium uptake protein 1*; IMS: intermitochondrial space; *PACS-2*: *Multifunction sorting Protein-2*; ER-Endoplasmic reticulum; PS: phosphatidylserine; PE: phosphatidylethanolamine; IMM: intermitochondrial membrane; *SERAC1*: *serine active site-containing protein 1*; *TANGO2*: *transport and Golgi organization 2 homolog*; OMM: outer mitochondrial membrane; del: deletion.

### Calcium Shuttling

#### Endoplasmic Reticulum—Mitochondria Calcium Cross Talk: Mitochondrial Calcium Uptake Protein 1 (MICU1)

Mitochondrial calcium hemostasis regulates many aspects of cellular physiology. Calcium is a major cellular secondary messenger and stored within the endoplasmic reticulum (Koch, 1990). Upon physiological stimulation, opening of inositol 1,4,5-triphosphate–gated channels of the ER exposes the mitochondria MAM to high concentrations of calcium (Filadi et al., 2017). Cell cultures have demonstrated rapid diffusion of calcium into the mitochondria across the close approximation between the two organelles and stimulate mitochondrial metabolism (Rizzuto et al., 1998). The MAM structure linking the ER inositol 1,4,5-triphosphosphate receptor, mitochondrial voltage-dependent anion channel 1, and chaperone glucose–regulated protein 75, make up the close juxtaposition of the two structures (Szabadkai et al., 2006; De Stefani et al., 2012). The mechanism of how the structures within specific MAMs recognize each other spanning the two organelles remains unclear. Multiple necessary calcium-regulated functions have been described. The increased mitochondrial calcium concentration is required for fission (Chakrabarti et al., 2018), autophagy (Hailey et al., 2010), production of ATP (Decuypere et al., 2011), and mitophagy (Alirol et al., 2006). After entry, the larger mitochondrial surface quickly diffuses calcium, which downregulates the calcium stimulation.

The scaffolding modulating the calcium MAM and ER–mitochondria interaction include the OMM protein, protein tyrosine phosphatase-interacting protein-51 (PTPIP51), and vesicle-associated membrane protein B in the ER (VAPB; De Vos et al., 2012). Modulation of the scaffold protein expression controls ER–mitochondrial contacts (Stoica et al., 2014; Gomez-Suaga et al., 2017). The pore-forming calcium uniporter complex within the calcium MAM scaffolding comprises mitochondrial calcium uniporter protein (MCU), mitochondrial calcium uptake protein 1 (MICU1), MCU regulator (EMRE also known as SMDTI), MICU2, and MCUb, with a preferentially expressed central nervous system–expressed protein MICU3 (reviewed in Kamer and Mootha, 2015). MICU1 and MICU2 in the IMS are linked to the EMRE and MCU in IMM. When calcium concentration within the IMS is low, MICU1 and MICU2 inhibit calcium uptake. When calcium concentration rises, the inhibition is relieved allowing calcium flow into the mitochondrial matrix. The exact function of the MCUb is unclear.

Case Report: Brothers aged 2 and 4 years both presented with global developmental delay and hypotonia. The parents and other two siblings are healthy. Both brothers were born at term with noted elevated alpha fetal protein levels. The older brother developed clinical symptoms of motor delay and extrapyramidal abnormal movements at 2 months of age. Early in the workup, he was noted to have elevated creatine kinase, 3,080 (35–230); AST, 149 (5–41); and ALT, 119 (5–31) indicating possible liver and muscle injury but normal levels of bilirubin, GGT, and PT (INR)/PTT. At 4 years of age, the older brother had no expressive language, was not walking independently but could sit and stand with help. At 2 years of age, the younger brother has just begun to sit independently. He also had elevated creatine kinase, 1,186 with elevated AST and ALT levels. As with the older brother, the bilirubin, GGT, and PT (INR)/PTT values were within the normal range. He had no expressive language and was only beginning to sit. Both brothers had intellectual disability, with expressive language most involved and muscle weakness with hypotonia. Both brothers had elevation of alpha fetal protein, which has not been reported before. Elevated levels suggest liver regeneration or types of cancers, but clinical presentation and analyte testing did not indicate that either was present. Currently, the presence of this protein elevation remains unclear.

**FIGURE 1:**
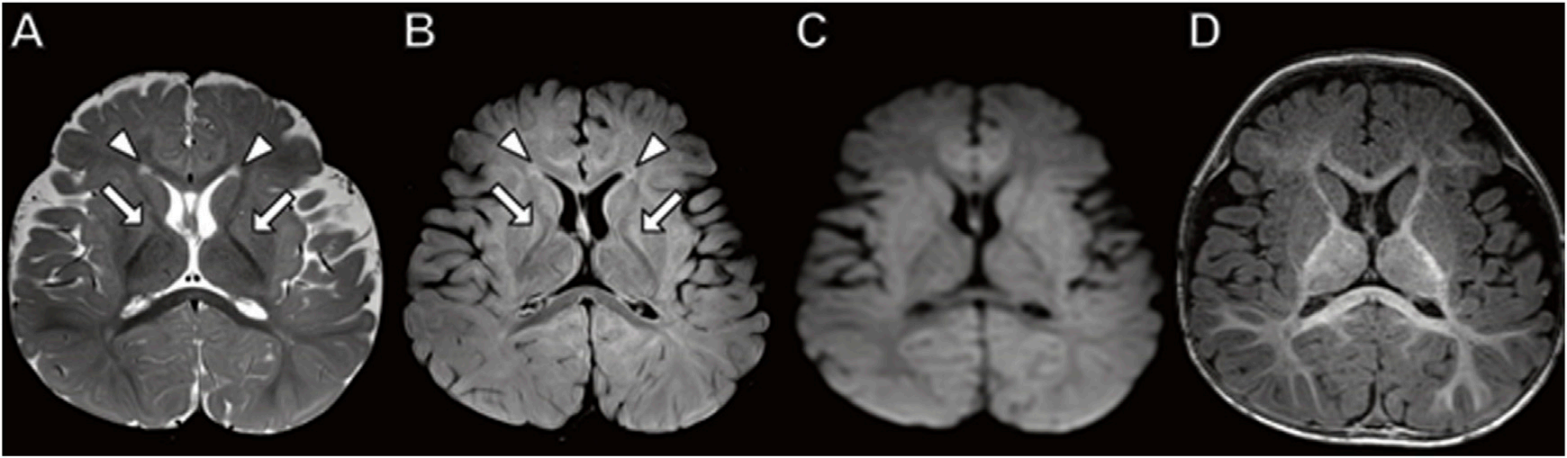
Brain MRI in 7-month-old boy with *MICU1* mutation. **(A)** Axial T2 and **(B)** axial fluid-attenuated inversion recovery (FLAIR) images demonstrate a mild signal hyperintensity in the globus pallidus (arrows) and frontal horn periventricular white matter (arrowheads). No abnormal signal on diffusion-weighted **(C)** or T1-weighted **(D)** images.

The older brother had a brain MRI at 7 months of age (Figure 1) showing mild equivocal T2-weighted and fluid-attenuated inversion recovery (FLAIR) signal hyperintensity in the frontal periventricular white matter and globus pallidus. There were no other structural brain abnormalities including no cortical malformations, normal cerebellum, and no abnormal calcifications.

The older brother underwent massive parallel gene sequencing (whole exome sequencing or WES) as did his parents. Biallelic heterozygous variants in the *mitochondrial calcium uptake 1* (*MICU1*) gene were found, c. 161 + 1 G>A (IVS2+1G>A) and c. 386 G>C (p. Arg29Pro). The same variants were found in the younger brother. The paternal inherited variant is a canonical splice site change which is likely pathological. The variant inherited from mother has not been described in the literature; however, an *in silico* analysis supports a deleterious effect. Neither of the two variants has been observed at significant frequency in large population cohorts (Lek et al., 2016).

The phenotype of both brothers is compatible with the limited reported patients in the literature. Two of the largest studies of *MICU1*-related disease together report only 30 patients (Logan et al., 2014; Musa et al., 2019). A total of 30 patients with onset in young childhood had early onset but static course of proximal muscle weakness with intellectual disability, elevated serum creatine kinase, and liver transaminitis. Most subjects have extrapyramidal motor signs that are progressive and a few with hypotonia. Abnormal signals in the globus pallidus (Logan et al., 2014) and periventricular white matter (Wilton et al., 2020) equivocally observed in our case has been previously reported on MRI. Other *MICU-1*-associated imaging phenotypes reported include a normal brain MRI (Lewis-Smith et al*.*, 2016); cortical, cerebellar and/or basal ganglia dysplasia (Wilton et al., 2020); and white matter calcifications (Logan et al., 2014). The creatine kinase and liver transaminase biochemical abnormalities observed in our cases is compatible with prior case reports (Logan et al., 2014; Musa et al., 2019).

Our two brothers demonstrate some variation in phenotype expression compared to the reported literature and between each other. For example, the younger brother has not developed the most observed extrapyramidal abnormal movements, which suggests phenotypic variation even with identical genetic variants.

As more patients are reported in the clinical literature, specific mechanistic findings will uncover how this disorder of calcium control may account for phenotypic variation. Mitochondrial calcium regulation of MICU1, MICU2, and EMRE through the calcium uniporter (MCU) MAM is central to multiple mitochondrial and cell functions. There is growing literature demonstrating that control of bioenergetics, spatiotemporal organization of calcium ion signals, and apoptosis are part of these functions (Shah and Ullah, 2020).

#### Regulation of ER–Mitochondria Communication: Maintenance of Mitochondrial Homeostasis; Multifunction Sorting Protein-2 (PACS-2)

Autophagy occurs universally to remove cellular components that accumulate during normal cell functioning. In addition, during development and following nutrient starvation, autophagy provides nutrients from their own intracellular components. During autophagy, damaged organelles and aggregated proteins are engulfed with specialized double-membrane vesicles known as autophagosomes. These vesicles then fuse with the endosomal lysosomes and are subsequently degraded to yield metabolites that can be released into the cytoplasm for recycling (Mizushima, 2007). The mitochondrial protein PTPIP51 and the ER protein VAPB act as tethers that regulate autophagy (Gomez-Suaga et al., 2017). Another essential role of autophagy is calcium homeostasis. Impaired calcium transfer can negatively affect ATP production resulting in increase of the cytosolic adenosine monophosphate to ATP ratio, which can lead to activation of AMP-activated protein kinase and autophagy (Decuypere et al., 2011; Thomas et al., 2017). Early studies have demonstrated that in starved cells the OMM participates in autophagosome biogenesis. OMM fragments are found in autophagosomes but IMM and mitochondrial matrix markers are not. Abnormal autophagy is thought to be a key factor in some diseases such as cancer and neurodegenerative disease (Rubinsztein et al., 2012).

Mitophagy is a distinct type of autophagy characterized by selective removal of damaged and unwanted mitochondria. It is essential for mitochondrial quality control, homeostasis, and preventing cell death (Kubli and Gustafsson, 2012). Alterations in mitochondrial morphology and function are observed during the early phases of apoptosis and are prerequisites for the initiation of mitophagy (Karbowski and Youle, 2003; Springer and Kahle, 2011). Mitophagy also takes place during specific developmental processes, such as erythrocyte maturation (Mortensen et al., 2010). Mitochondrial fusion and fission are involved in organelle integrity by sequestering damaged mitochondria for removal. The cellular mechanism distinguishing autophagy and apoptosis from mitophagy signaling is not completely clear at present. One mechanism tying the two processes to the MAM has been the elucidation of multifunction sorting protein-2 (PACS-2). Simmen et al. (2005) described this protein to be involved in the formation of ER lipid-synthesizing centers found on MAM. When PACS-2 is depleted, there is mitochondrial fragmentation and uncoupling from the ER, induced apoptosis, and initiation of the cell death spiral (Simmen et al., 2005). By an unknown mechanism, selective PACS-2 depletion promotes mitochondrial homeostasis by increasing fission without apoptosis (reviewed by Killackey et al., 2020). How PACS-2 depletion can be selective and induce mitochondrial homeostasis and the separate process of apoptosis remains unclear.

Case Report: A 5-month-old boy presented with neonatal onset of seizures, mild dysmorphic features, and developmental delay. Delivery was at term via cesarean delivery when he was noted to be hypotonic with high arched palate with micrognathia, muscular cardiac ventricle septal defect, and micropenis. Due to poor suck and difficulty with latching on, he required a feeding tube soon after birth. Seizures were noted at day 5 of life with abnormal electroencephalogram (EEG) demonstrating focal seizures arising independently from the left and right hemisphere. Antiseizure medication, phenobarbital and levetiracetam were initiated with oxcarbazepine replacing levetiracetam. At discharge (day of life 14 days) he was seizure free, and his feeding had progressed to fully oral intake. As time progressed, the phenobarbital was weaned off, but oxcarbazepine was needed to control seizure activity.

His endocrinology workup was normal and testosterone therapy was initiated for his micropenis. His muscular cardiac ventricle septal defect closed on its own. Soon after delivery, his parents noticed difficulty in hearing, and he was diagnosed with bilateral sensorineural hearing loss. He developed food protein induced enterocolitis syndrome due to allergies to egg, peanut, and wheat. Developmentally, he was delayed in gross motor skills and did not walk independently until 26 months of age. Fine motor skills were also delayed with inability to feed himself, which was not attained until over 2 years of age. At 26 months of age, he knew about 20 words but could not combine words. His Early Learning Composite was 65 falling into the impaired range. He was diagnosed with autistic spectrum disorder at age 2.

**FIGURE 2:**
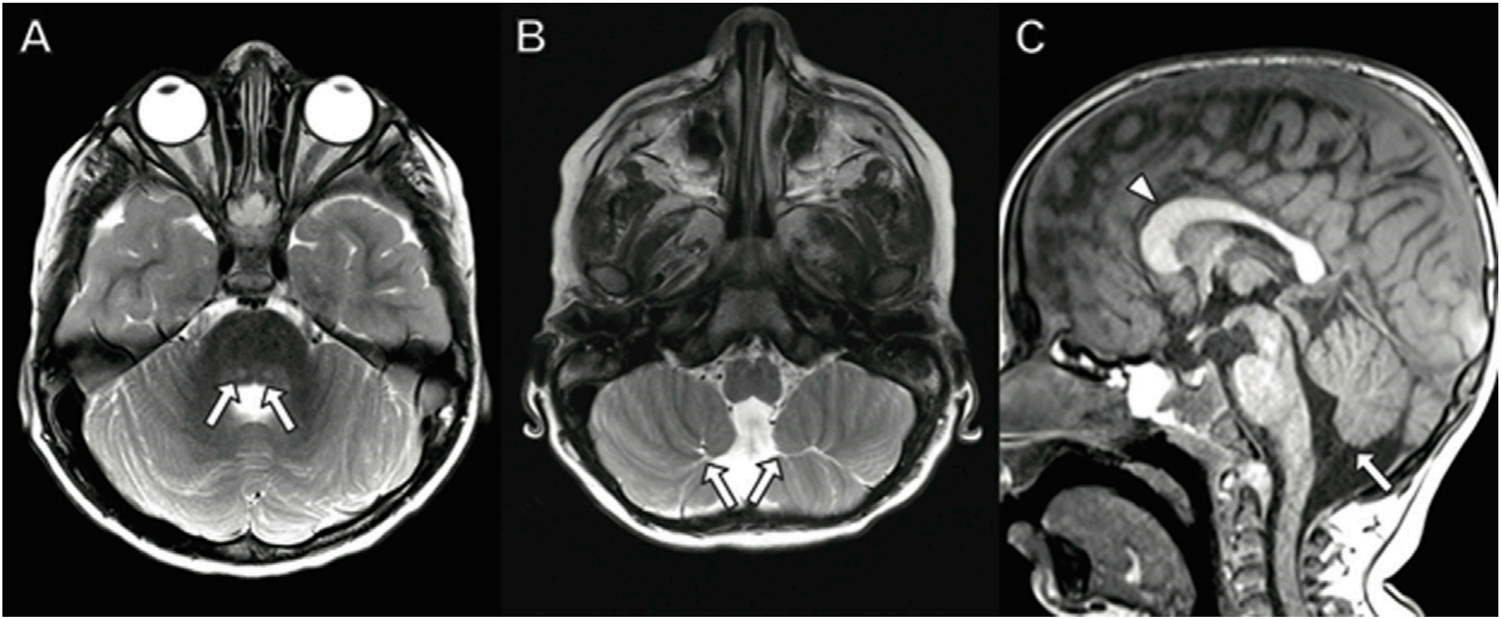
Brain MRI in a 3-year-old boy with *PACS2* mutation. **(A)** Axial T2 demonstrates hyperintense signal in the central tegmental tracts (arrows). **(B)** Axial T2 image shows dysplastic cerebellar folia (arrows). **(C)** Sagittal T1 image with dysplastic, abnormally thick genu of the corpus callosum (arrowhead) and vermian hypoplasia (arrow).

Brain MRI performed at 3 years of age (Figure 2) demonstrates dysplastic cerebellar folia, mild cerebellar vermian hypoplasia, thickened genu of the corpus callosum, hippocampal inversion, and T2 hyperintense signal in the central tegmental tracts. No cerebellar cysts were present. The hypothalamus, pituitary, and orbits were normal.

Genetic testing was performed using massive parallel gene sequencing of the genome (WES) trio testing which includes the patient and both parents. He was found to have a *de novo* heterozygous variant in the *PASC-2* gene, c. 625 G>A (p. Glu209Lys). This variant has not been observed in large population cohorts and in silico analysis predicts a deleterious effect (Lek at al., 2016).

There have been 14 individuals possessing the c. 625 G>A variant and a single individual with a novel variant c. 631 G>A (p. Glu 211Lys) previously described in the literature, all occurring *de novo* (Olson et al., 2018; Dentici et al., 2019). Thirteen of the 15 individuals had neonatal-early infantile onset seizures with developing cognitive delay, developmental and epileptic encephalopathy (DEE) as observed in our case. Facial dysmorphism was common including synophrys, hypertelorism, down slanting palpebral fissures, bulbous nasal tip, wide mouth with downturned corners and thin upper lip. Our patient had mild plagiocephaly due to hypotonia. He also had a high arched palate with mild micrognathia. However, compared to the other reported cases, he did not have the other reported facial dysmorphisms.

The reported neuroimaging phenotype associated with *PACS-2* mutations is most commonly cerebellar dysplasia including abnormal foliation and vermian hypoplasia as observed in our case (Olson, H. E. et al., 2018, Dentici et al., 2019). Less commonly associated findings include dysplastic corpus callosum, hypothalamic fusion anomalies (Olson, H. E. et al., 2018), low white matter volume (Dentici et al., 2019), and coloboma (Sakaguchi, Y. et al., 2021) which were not observed in our case.

#### Phospholipid Remodeling: Serine Active Site-Containing Protein 1 (SERAC1)

The origin of most lipids imported into mitochondrial occurs via the ER. Phosphatidylglycerol (PG), cardiolipin, and phosphatidylethanolamine are synthesized within mitochondria but phosphatyidylcholine, phosphatidylinositol, phosphatidylserine, and sterols are imported into the mitochondria by a specific MAM. This MAM has been found to contain a number of lipid synthesizing enzymes that are critical for synthesis and shuttling of lipids from the ER to mitochondria (Gaigg et al., 1995; Vance, 2014). There is growing evidence for interplay between the phosphatidylethanolamine and cholesterol metabolism in motor neuron disease phenotypes (Rickman et al., 2020).

The serine active site-containing protein 1 (SERAC1) involved in phosphatidylglycerol remodeling is essential for mitochondrial function and intracellular cholesterol trafficking (Sequeira et al., 2017). SERAC1 contains a C-terminal serine-lipase/esterase domain with a consensus lipase motif located at the MAM (Wortmann et al., 2012). Abnormal function can increase the ratio of phosphatidylglycerol-34:1 to phosphatidylglycerol-36:1 and decrease the level of mono-acyl-glycerol (bis) phosphate and subsequently altering the accumulation of cholesterol (Wortmann et al., 2012). Recently, *SERAC1* pathological variants were also found to change acyl-chain composition of PG, a precursor of cardiolipin, giving rise to normal levels of cardiolipin but possessing altered acyl chain composition (Snanoudj et al., 2019).

Case Report: A term girl infant presented with hypotonia, low birth weight (15%), short length (5%) and microcephaly (Z score −9.3). She failed her newborn hearing screening evaluation and subsequent Auditory Brainstem Response testing suggesting a moderate sensorineural hearing loss in the right ear and severe loss in the left ear. She was fitted with hearing aids at 6 months of age. Despite being hypotonic and microcephalic at birth, her development seemed on track until 5–6 months. At that time, she was beginning to sit independently and beginning to use her elbows to crawl. However, she began to express motor regression losing her ability to hold objects with either or both hands. At 12 months she could eat orally but her mother noted difficulty in chewing. At 27 months of age, her verbal skills were delayed with no expressive language, and she stopped making consonant sounds; moreover, she remained short statured with length at 8.5% and weight at 0.3%.

At 12 month, a laboratory analysis revealed an elevated alpha fetal protein of 122 (<10 mg/g creatine), mild elevation of 2-ketoglutaric of 233 (<153 mg/g creatine) and 3-methylglutaric acid of 26 (<10 mg/g creatine). These labs suggest liver hepatopathy and oxidative phosphorylation defect. She underwent a liver biopsy at 13 months which demonstrated mild portal fibrosis and scattered portal septae. There was no evidence of tumor and over the next several months, AFP levels normalized.

**FIGURE 3:**
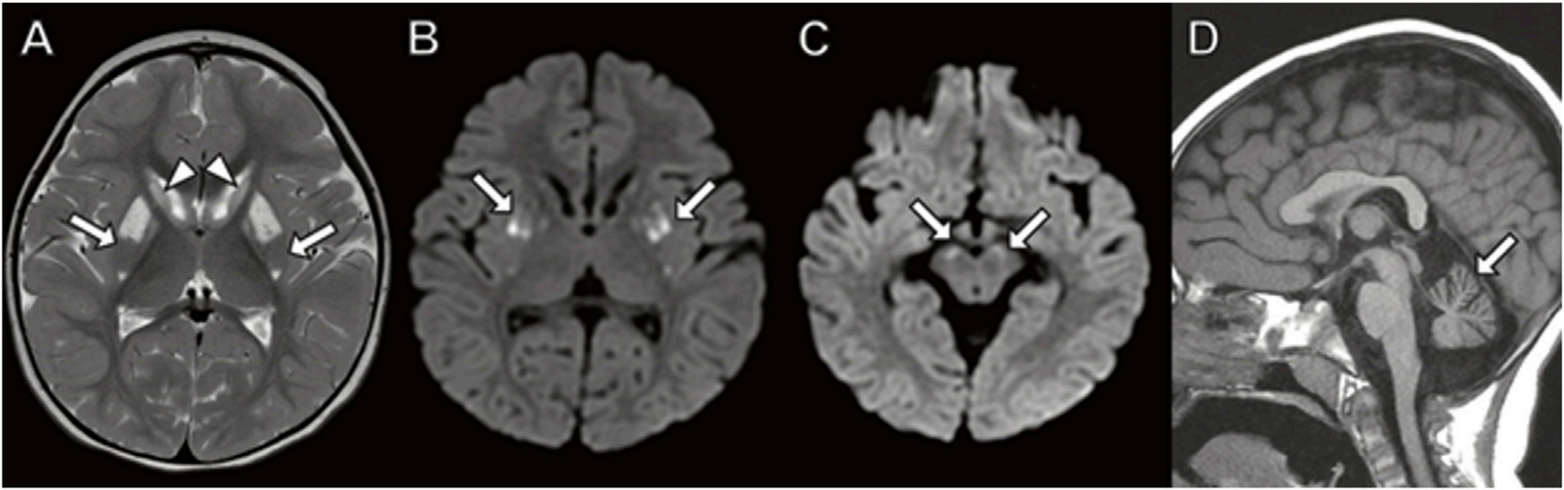
Brain MRI in 22-month-old girl with *SERAC1* mutation. **(A)** Axial T2 image demonstrates abnormal signal throughout the basal ganglia with sparing of the dorsal putamen (arrow) resulting in a finding described as the “putaminal eye”. There is abnormal signal and atrophy of the caudate heads (arrowheads) and globus pallidus. **(B,C)** Axial diffusion-weighted images with restricted diffusion in the putamen (**(B)** arrows) and substantia nigra (**(C)** arrows). **(D)** Prominent cerebellar folia indicating cerebellar atrophy (arrow) is present on sagittal T1-weighted image.

At 22 months of age, she underwent neuroimaging with MRI demonstrating bilateral T2 signal hyperintensity in the putamen with a spared dorsal region consistent with a “putaminal eye” (Figure 3), typical of the disorder 3-methylglutaconic aciduria with deafness–dystonia, hepatopathy, encephalopathy, and Leigh-like syndrome (MEGDEL; Wortmann et al., 2015). Moreover, the MRI demonstrated smaller areas of diffusion weighted imaging (DWI) signal abnormality (reflecting cytotoxic edema) involving the caudate, putamen, globus pallidus, and substantia nigra. There was atrophy of the caudate, globus pallidus, and cerebellum.

Our patient had an older brother who was diagnosed with sensorineural hearing loss soon after birth. He never developed expressive language and did not sit independently or walk. At about 1.5 years of age, he stopped oral feeding and required G-tube for nutrition. At that time, he underwent analyte testing and was found to have moderated elevations of 3-methylglutaconic acid in his urine. He passed away at age 3 years. Genetic testing and MRI were not performed.

About the time of the liver biopsy, the family underwent trio massive parallel gene sequencing (WES) of the genome and our patient was found to have homozygous biallelic variants in the *SERAC1* gene, c. 1763del T (p. Val 588 Gly FsX11). Most pathological variants in SEARC1 have been frameshift, nonsense, or missense changes within or upstream of the serine-lipase domain and uncommonly on the carboxy terminal side of the serine-lipase domain (Wortman et al., 2012; Sarig et al., 2013; Wortman et al., 2015). Her homozygous biallelic variants in the carboxy terminal of *SERAC1* have not been previously reported. We feel this represents a new pathological variant as an etiology for this syndrome. To date, this variant has not been validated as pathological, but based on our patient’s clinical phenotype and labs we predict this variant is likely disease-inducing. WES sequencing of the mitochondrial genome was without abnormality.

Our patient fits the clinical, laboratory, and neuroimaging phenotypes described for MEGDEL syndrome due to mutations in *SERAC1*. The most common onset of MEGDEL is during infancy presenting with hypoglycemia and sepsis-like disease followed by delayed psychomotor development apparent at about 2 years of age with sensorineural deafness, spasticity, and excretion of 3-methylglucoconic acid (Maas et al., 2017; Sequeira et al., 2017; Fellman et al., 2022). Our patient had early onset neurosensory hearing loss and global developmental delay. About 50% also have severe liver involvement, as in our case, with earlier onset patients having features of liver failure of elevated transaminitis and conjugated bilirubin. Beyond the neonatal period patients do not express features of liver failure but liver involvement can be seen. The elevation of alpha fetal protein in our case has not been reported in the MEDGEL literature. Our case also demonstrates mitochondrial dysfunction that often reported in this disorder. Unfortunately, current treatment is supportive with 50% of patients surviving into the teenage years (Maas et al., 2017).

Brain MRI in patients with MEGDEL classically demonstrates T2/FLAIR and DWI signal abnormality in the basal ganglia, including substantia nigra, which evolves in a pattern associated with clinical worsening (Wortmann et al., 2015). The initial change in the basal ganglia occurs with the first neurological signs, with palladium involvement. This is followed by edema of the caudate nucleus and putamen with sparing of the mid-dorsal putamen, named the “putaminal eye.” This finding is thought to be pathognomonic for this syndrome. Most show end stage basal ganglia disease with atrophy by 3 years of age, at the time of onset of dystonia. Other reported neuroimaging findings include cerebellar atrophy, as observed in our case, and thin corpus callosum (Alagoz, M., et al., 2020).

#### Alteration of Fatty Acid–Metabolizing Enzymes: CYP2U1 Protein

MAMs are enriched in cholesterol, phosphatidylethanolamine and lipases which are involved in biosynthesis, and import of phosphatidylserine decarboxylase pathway in mitochondria. (Vance, 2014). The MAM serve as a conduit through which lipidome pathways may converge and interact with phosphatidylethanolamine and cholesterol metabolic pathways (Rickman et al., 2020). Defects in these pathways can lead to upper and lower motor neuron disease. Hereditary spastic paraplegias are a group of motor neuron diseases that are clinically heterogeneous inherited neurodegenerative disorders. Clinically, these disorders are characterized by slowly progressive lower-limb spasticity and weakness that worsen over time because of corticospinal-tract degeneration (Harding, 1983; Fink, 2003).

CYP2U1 is a cytochrome P450 protein implicated in ω− and ω−1 fatty acid hydroxylation of arachidonic acid and related long-chain fatty acids (Chuang et al., 2004). The exact functions of CYP2U1 remain unclear but is a structurally related member of the CYP2 gene family. Cytochrome P450 hemeproteins are mostly involved in oxidative metabolism of xenobiotics such as drugs, environmental chemicals or endogenous steroid hormones, fatty acids, eicosanoids, and vitamins (reviewed in Guengerich, 2015). Approximately 25% are “orphan” as their cellular function remains unknown (Stark and Guengerich, 2007). CYP2U1 belongs to this orphan group. It is highly expressed in brain and thymus but found in most tissues (Bieche et al., 2007). Western blot analysis demonstrated protein in microsomes and mitochondria (Dutheil et al., 2009). It is hypothesized that CYP2U1 is located within the IMM and involved in the decarboxylation of phosphatidylserine into phosphatidylethanolamine (Rickman et al., 2020). Homozygous or compound heterozygous pathological variants in *CYP2U1* have been shown to induce spastic paraplegia-56 (SPG-56; Tesson et al., 2012).

Case Report: This child was the product of a normal pregnancy and term delivery. He developed normally for the first 6–7 months, sitting independently, and attempting to pull to a stand. The parents subsequently noted regression as he stopped sitting with associated lower extremity “stiffness” and decreased movement. Moreover, he had increased startles to quick movements. Neurological exam at 7 months of age demonstrated central hypotonia. His workup at the time revealed a normal MRI of the brain (not shown) and analyte testing. Only generalized background slowing for age was noted on an electroencephalogram (EEG). By 12 months of age, he lost the ability to roll over. Over time, his lower extremities began to demonstrate frank spasticity, with muscle cramping. He suffered loss of fine mother skills, particularly loss of pincher grasp and the ability to hold objects in his hands. Slowly, spasticity evolved to include his upper extremities, although less involved than the lower extremities. He subsequently developed dysphagia and by 5 years required a gastrointestinal tube for nutrition. He underwent correction surgery at age 9 years, and a baclofen pump was inserted for spasticity of his upper and lower extremities. He developed neuromuscular scoliosis which required surgical correction at 14 years. At 17, his ophthalmology exam revealed right macular dystrophy. He had undergone muscle biopsy at age 10 and enzymatic testing of the electron transport chain demonstrated pathologically low complex I activity that was 17.7% of control values (Table 2; Bernier et al., 2002). Electron microscopy finding showed disruption of the sarcomeres with scattered fibers with sarcoplasmic masses with accumulations of granular material, small mitochondria, and disrupted myofilaments beneath the sarcolemma.

**TABLE 2:** Spectrophotometry analysis of electron transport chain enzyme activity from muscle biopsy material. These results are from the muscle biopsy of the patient with *CYP2U1* gene variants.

Complex	Activity	Control Range (n=49)	Percent Mean
Complex I	0.2	1.2 + 1.1 (range = 0.2–4.7)	17.5 %
Complex II/III	3.0	2.1 + 12.9 (range = 0.5–4.9)	138.8 %
Complex II	1.0	0.8 + 0.4 (range = 0.1–2.0)	119.9 %
Complex III	21.0	15.2 + 6.8 (range = 6.8–35.2)	137.6 %
Complex IV	114.3	148.9 + 67.2 (range = 57.3–373.0)	76.7 %
Citrate synthase	25.6	18.6 + 4.7 (range = 9.4–30.0)	137.5%

Activity (μmole/min/g wet weight); Complex I represents NADH-cytochrome c reductase (rotenone sensitive); Complex II/III, represents NADH-ferricyanide reductase; complex II, represents succinate dehydrogenase; Complex III, represents decylubiquinol-cytochrome c reductase; Complex IV, represents cytochrome c oxidase.

**FIGURE 4:**
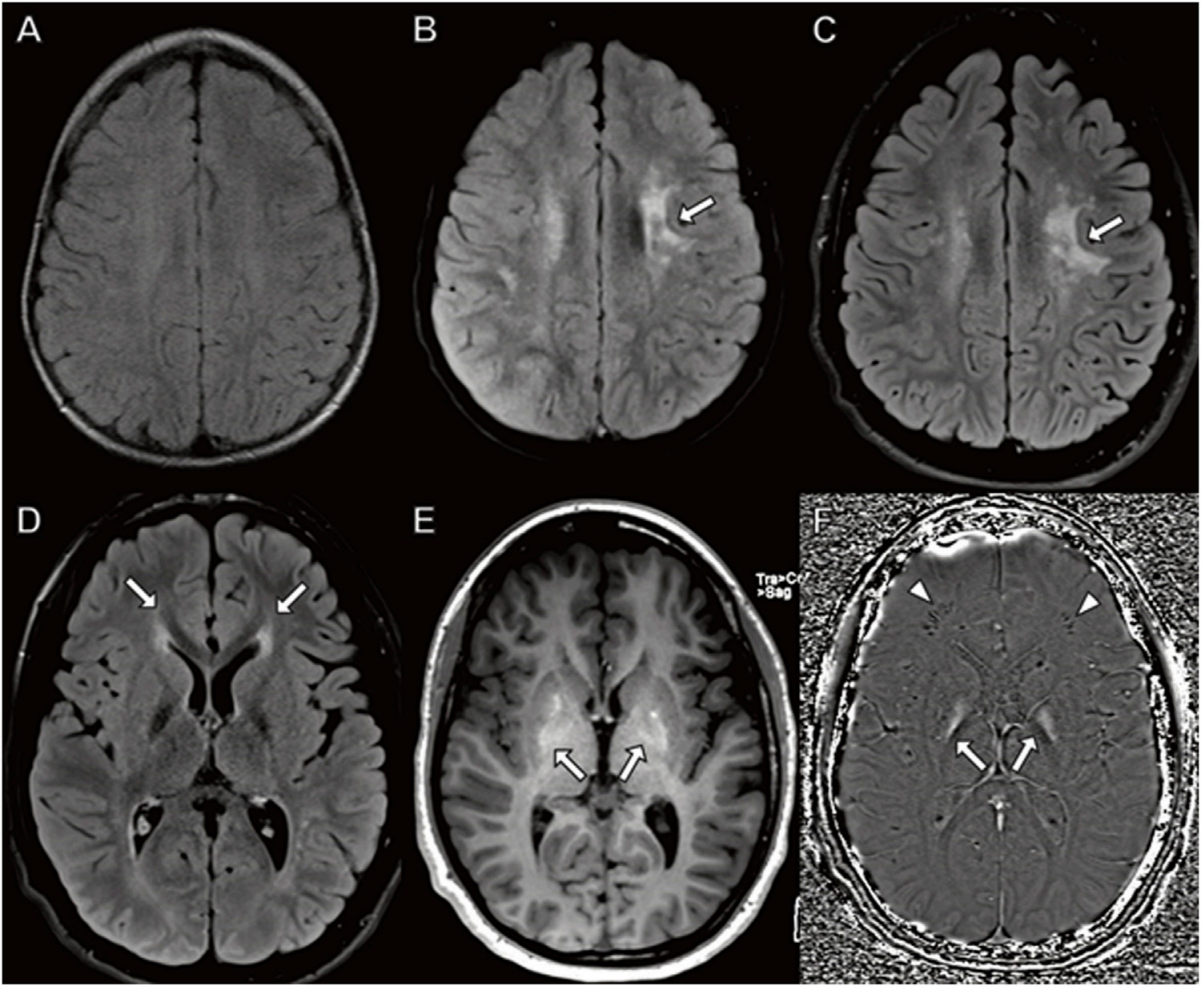
Brain MRI in a boy with *CYP2U1* mutation. **(A–C)** Axial fluid-attenuated inversion recovery images at **(A)** 3 years **(B)** 9 years, and **(C)** 15 years demonstrate worsening, patchy hyperintense signal in the centrum semiovale involving subcortical U fibers (**(B,C)** arrows). **(D–F)** MRI at 15 years with **(D)** abnormal hyperintense signal extending from the frontal horns of the lateral ventricles reminiscent of the “ear of the lynx” sign (arrows). **(E)** Abnormal signal hyperintensity in the globus pallidus on axial T1-weighted images corresponding to **(F)** hyperintense signal on axial susceptibility-weighted phase image (arrows) indicating mineralization. **(F)** Linear hypointense signal in the frontal white matter representing calcifications in a perivenular distribution (arrowheads).

Although his initial brain MRI at 7 months was normal (not shown), follow-up MRI studies at 3.5, 9 and 15 years (Figure 4) demonstrated worsening patchy abnormal T2/FLAIR hyperintense white matter signal in a frontoparietal predominance with associated progressive atrophy and corpus callosal thinning. The abnormal white matter signal at the frontal horns of the lateral ventricles is suggestive of the “ear of the lynx” finding previously reported (Tesson, C. et al., 2012). In addition, there was abnormal signal in the globus pallidus representing calcification and/or mineralization. Abnormal white matter calcification in a perivenule distribution was also present.

When commercial gene testing became available, trio massive parallel WES gene sequencing of his mother, father and our patient demonstrated biallelic heterozygous variants in *CYP2U1*, c. 342 C>A (p. Tyr114Ter) and c. 1,001 del A (p. AsnThrfxX37). Both variants are predicted to result in protein truncation or nonsense mediated decay, which is known mechanism of disease. Neither variant has been observed at a significant frequency in large population cohorts (Lek et al., 2016). WES of the mitochondrial genome was normal. Given the phenotype of our patient, we feel the variants are pathological and fit the SPG56 syndrome of hereditary spastic paraplegia (HSP). His biochemical workup and structural location of CYP2U1 protein suggest a primary mitochondrial disorder.

To date, just over 30 patients have been reported in the literature with *CYP2U1* variants leading to SPG 56 (Sharawat et al., 2021). In total, 22 patients had homozygous and 5 had compound heterozygous variants (Masciullo et al., 2016; Bibi et al., 2020; Sharawat et al., 2021). The median age of symptom onset was 1 year (range 1–3 years), with neurological symptoms in over 50% between 9 months and 1 year. Approximately 40% were able to walk during the onset of symptoms with lower and upper extremity being universal. Our patient fits the most common presenting symptoms and progression of lower and upper extremity involvement. Intellectual disability was noted in almost 60%. However, there is a wide range of onset, with some patient symptoms only beginning well into the third decade (Leonardi et al., 2016). Our patient developed early onset macular dystrophy at age 17 years, well before the published cases. Reported neuroimaging findings associated with *CYP2U1* mutations include abnormal white matter signal including surrounding the frontal horns of the lateral ventricle (“ear of lynx”), thin corpus callosum, and abnormal globus pallidus signal similar to that observed in our case (Tesson, C. et al., 2012). Less commonly reported findings not observed in our case include dorsal hydromyelia and mild atrophy of the brainstem and cerebellum (Minase, G. et al*.* 2017; Tesson et al., 2012; Masciullo et al., 2016). In some cases, brain MRI is reportedly normal (Masciullo, M. et al., 2017; Citterio, A. et al., 2014; Minase, G. et al., 2017). Our patient expands the phenotype and genotype and documents primary mitochondrial dysfunction in SPG 56 with this genotype.

#### Mixed Golgi and Mitochondrial Endoplasm Reticulum Transport: Transport and Golgi Organization Homolog 2 Protein

The TANGO2 (Transport and Golgi Organization 2 homolog) protein is without a precise localization or function in humans, but when altered, TANGO2 induces disease. *TANGO2* is highly conserved across all mammalian species. The original functional assays in a Drosophila S2 cell line localized the TANGO2 protein to the cytosol and Golgi membrane (Bard et al., 2006). Microscopic analysis demonstrated that depletion of TANGO2 protein led to fusion of Golgi and ER membranes. Multiple studies have found clinical similarity to disorders of long-chain fatty acid metabolism in patients with TANGO 2 deficiency, but without hypoketosis during metabolic decompensation (Lalani et al., 2016; Berat et al., 2021). Also, like patients with fatty acid disorders, metabolic decompensation seems only present during stress situations such as fasting or illness. Both the human and murine orthologue of *TANGO2* have a mitochondrial targeting sequence suggesting possible mitochondrial localization (Bard et al., 2006; Maynard et al., 2008). Furthermore, biochemical analysis of *TANGO2* pathological variant patient fibroblast cultures has demonstrated compromised palmitate-dependent mitochondrial respiration (Jennions et al., 2019). However, the variability of patient cell line findings in analytes, oxidative phosphorylation, mitochondrial fatty acid oxidation, and processing of secreted and plasma membrane proteins suggests the severity of mitochondrial compromise might dictate *TANGO2*-mediated disease expression (Jennions et al., 2019; Mingirulli et al., 2019; Berat et al., 2021).

Case Series: Family 1, the affected individual was a normal healthy young girl until about 16 months of age. During a trip she had a vomiting episode in her car seat. Due to ongoing lethargy, she was taken to the ED, and found to be dehydrated and hypoglycemic with a venous blood pH of 6.87 and CO2 of 39. The serum lactate was normal. The electrocardiogram was normal. Upon metabolic correction, she remained limited in responsiveness. Although she had no fever, she had an extreme leukocytosis with white cell count of 60.1 K/mm3.

Her EEG at presentation initially demonstrated a suppression burst pattern with generalized periodic pattern. Over the next several days, the periodic EEG pattern became intermixed with independent lateralized periodic pattern over the left and right hemisphere. We considered this pattern a non-convulsive status epilepticus (Fung et al., 2021). Epochs of status epilepticus, defined as greater than 50% seizure burden in a single hour, were finally controlled with a combination of multiple antiseizure medications after 16 days. She had persistent periodic subclinical seizures over the next 2 months, captured on continuous video-EEG monitoring. Over the period of the next 3 weeks, subclinical seizures became less frequent, and she began having seizure free days. The EEG studies showed multifocal epileptiform discharges over the left and right hemisphere with background slowing, without normal stage cycling. She began having clinical manifestations of seizures noted to be myoclonic, focal tonic, and secondarily tonic clonic seizures. She remains with episodic seizures.

**FIGURE 5:**
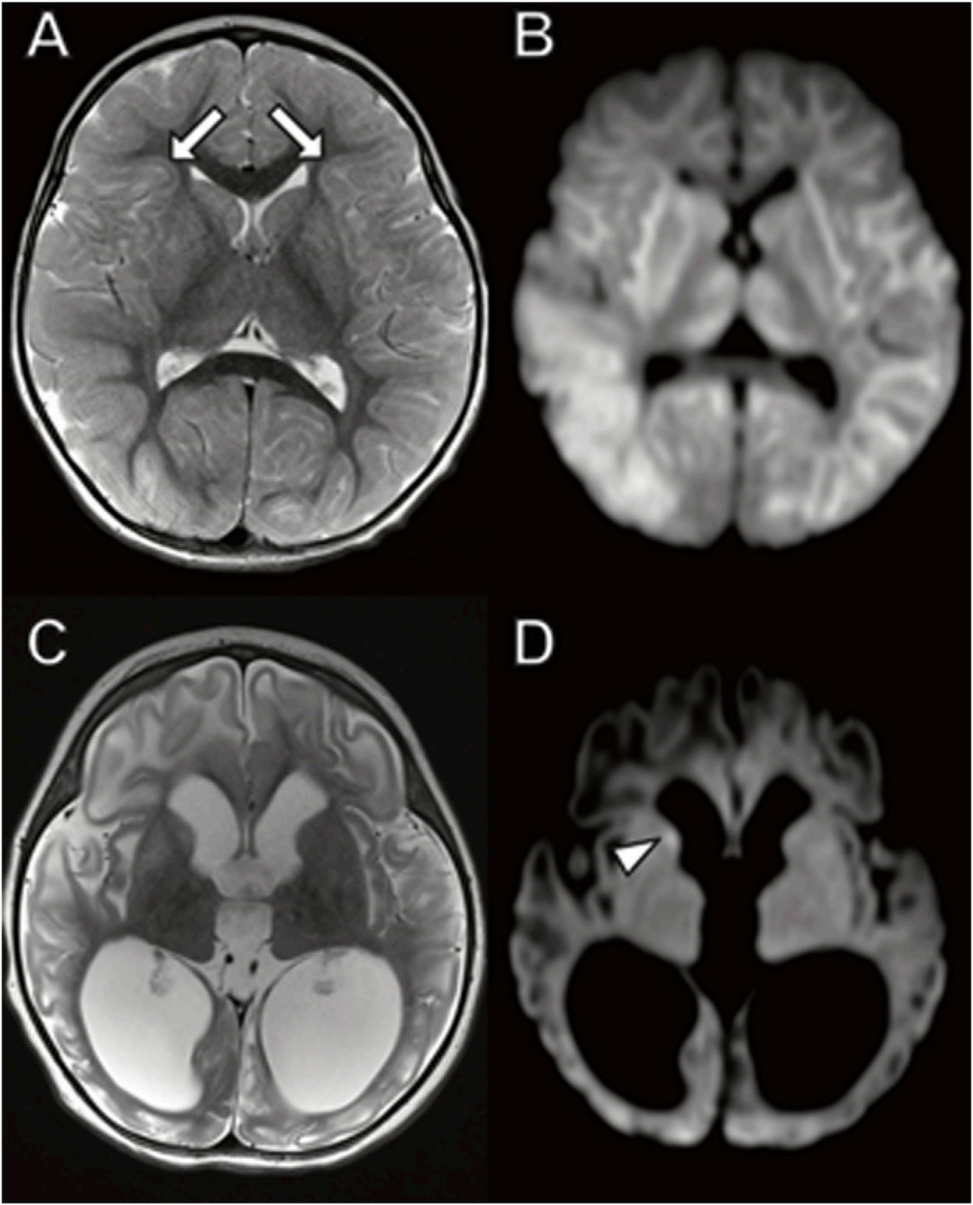
Brain MRI in young girl with *TANGO2* mutation. **(A,B)** Diffusely abnormal brain MRI at 16 months characterized by **(A)** abnormal hyperintense T2 and **(B)** diffusion-weighted imaging signal throughout the white matter and basal ganglia, including involvement of subcortical U fibers (A, arrows). **(C,D)** Follow-up brain MRI 2 months later with ventriculomegaly due to atrophy with **(C)** associated abnormal T2 signal hyperintensity involving cerebral white matter and basal ganglia. **(D)** Restricted diffusion is largely resolved with small area of persistent diffusion-weighted signal abnormality in the right caudate (arrowhead).

The day after her admission an MRI scan demonstrated widespread DWI and T2 signal hyperintensity involving the thalami, basal ganglia, and throughout the white matter including subcortical U fibers indicating cytotoxic edema (Figure 5). A large lactate peak was present on MR spectroscopy of the basal ganglia (not shown). The cerebellum was relatively spared. Ten weeks later a repeat study showed diffuse atrophy with abnormal T2/FLAIR signal in the white matter and deep gray structures.

The cardiac ECHO and ECG studies done at various times during the prolonged hospital stay were all normal. Liver transaminases have remained slightly elevated at 39–69 IU/L (normal: 5–31) for ALT and 48–146 IU/L (5–41) for AST. For the first week of the admission, serum lactate was elevated, 3.0–4.2 mmol/L (normal <2.1) and then returned and remained normal. Thyroid stimulating hormone reached a high of 52.1 (mcIU/mL (0.6–5.6 mcIU/mL) in the first week and was treated with thyroid hormone. Bilirubin, creatine kinase, Troponin, and free and total carnitine levels remained normal throughout the hospital stay. On trio massive parallel gene sequencing (WES), biallelic homozygous pathological variants involved the deletion of exons 3 through 9 in *TANGO2*. She remains in the hospital at 6 months after presentation in a stable but very guarded condition, with ongoing episodic seizures and severe cognitive and motor dysfunction.

**FIGURE 6:**
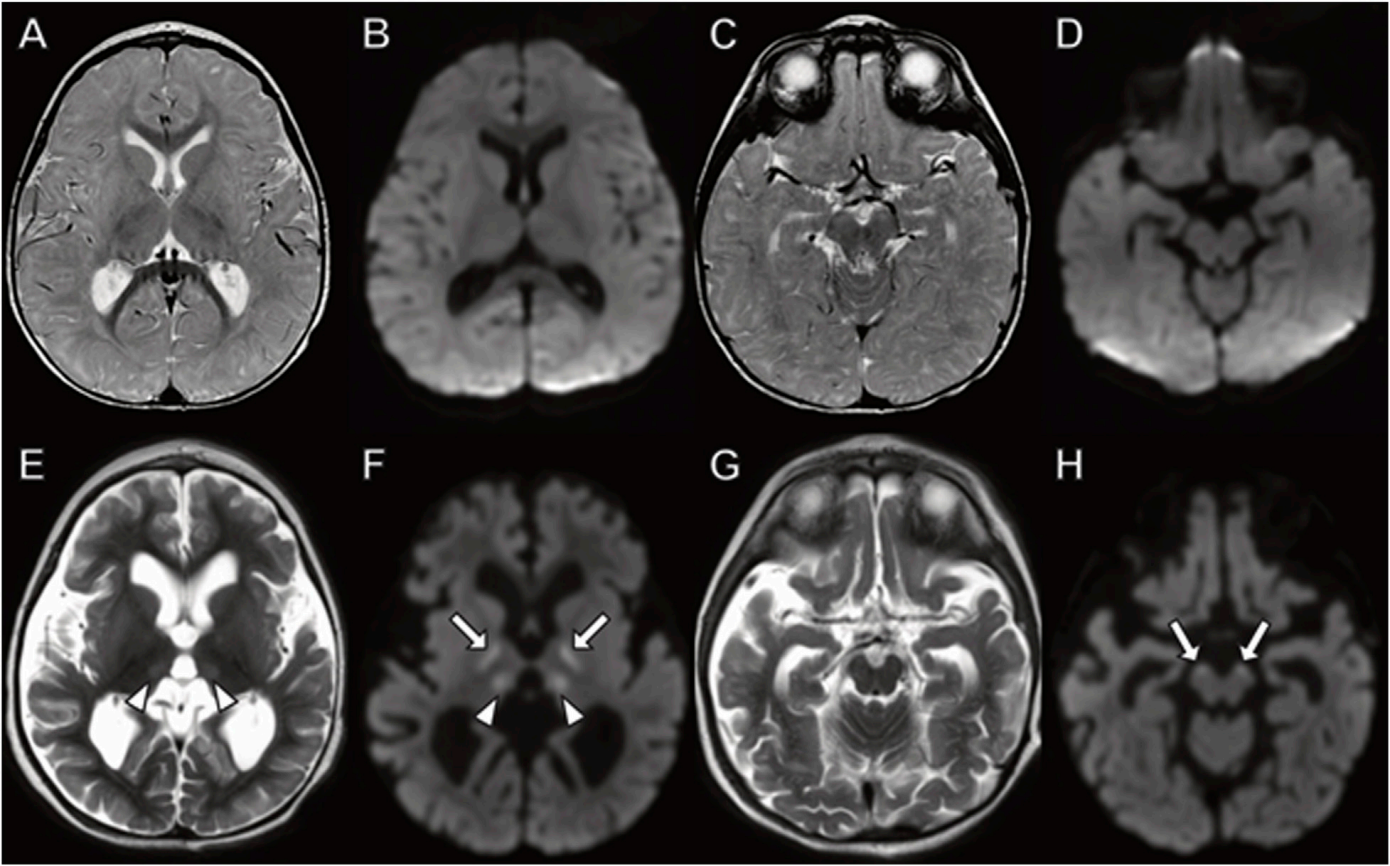
Brain MRI in boy with *TANGO2* mutation at **(A–D)** 8 months and **(E–H)** 15 months of age. The brain MRI at 8 months demonstrates no abnormal signal on **(A,C)** fluid-attenuated inversion recovery or **(B,D)** diffusion-weighted images. **(E–H)** At 15 months, diffuse cerebral atrophy is present with abnormal **(E)** T2 signal and **(F)** diffusion-weighted imaging hyperintensity in the thalamus (arrowheads). Additional areas of restricted diffusion involving the corticospinal tracts on diffusion weighted imaging (**(F,H)**, arrows).

In Family 2, the three siblings were briefly described 4 years ago (Dines et al., 2019). The index case of the second family was the oldest boy who was born at term and initially met developmental milestones. At 6 months of age, he developed poor feeding and regressed in motor skills. Within a month he stopped tracking and developed eye nystagmus with optic atrophy. His initial brain MRI and EEG study were normal. His MRI repeated at 15 months of age demonstrated cerebral, but not cerebellar atrophy, with DWI signal abnormality indicating cytotoxic edema in the thalamus and corticospinal tracts (Figure 6).

Due to failure to thrive, a nasogastric feeding tube was placed at 9 months of age with subsequent G-tube placement. He was noted to have increased tone in his lower extremities but central hypotonia. At 12 months he developed dystonic posturing of the upper extremities and the syndrome of infantile spasms, with EEG showing hypsarrthymia and epileptic spasms. Although epileptic spasms were successfully treated, he evolved into medically intractable tonic and myoclonic seizures. Seizure freedom did not occur; however, seizure intensity and frequency significantly reduced with valproic acid therapy. Over time, he developed limb spasticity. Unexplained treatment resistant nightly vomiting began occurring with weight stagnation. After the detection of renal stones, an endocrine workup found severe hypothyroidism.

To elucidate the etiology of his clinical picture a muscle biopsy was preformed which demonstrated a reduction of complex I (Table 3). Genetic testing revealed biallelic heterozygous pathological variants in *TANGO2*, including the common exon 3 through 9 deletion and a novel variant c. 711-3C>G. The latter variant is a splice site change and RNA expression studies revealed an insertion of 52 amino acids into the protein sequence before ending at a premature stop codon (NM_152,906.6 p. R237ins*53: Dines et al., 2019). He died at 3 years of age following initiation of comfort care in the setting of his developmental and epileptic encephalopathy and increasing abdominal discomfort a progressive weight loss.

**TABLE 3:** Spectrophotometry analysis of electron transport chain enzyme activity from muscle biopsy material. These results are from the muscle biopsy from the patient with *TANGO2* gene variants.

Complex	Activity	Control Range (n=49)	Percent Mean
Complex I	0.4	1.2 + 1.1 (range = 0.2–4.7)	30 %
Complex II/III	1.4	2.1 + 12.9 (range = 0.5–4.9)	65 %
Complex II	3.2	0.8 + 0.4 (range = 0.1–2.0)	400 %
Complex III	43.3	15.2 + 6.8 (range = 6.8–35.2)	284 %
Complex IV	91.3	148.9 + 67.2 (range = 57.3–373.0)	61 %
Citrate synthase	20.6	18.6 + 4.7 (range = 9.4–30.0)	111%

Activity (μmole/min/g wet weight); Complex I represents NADH-cytochrome c reductase (rotenone sensitive); Complex II/III, represents NADH-ferricyanide reductase; complex II, represents succinate dehydrogenase; Complex III, represents decylubiquinol-cytochrome c reductase; Complex IV, represents cytochrome c oxidase.

The younger brother and sister were found to have the same biallelic variants. However, both had less severe phenotypes compared to their older brother. Currently, the younger brother is 7-year-old, and his sister is now 5 years of age. The younger brother’s OFC at 4 months was 13% but at 2 years was <1% (>-2.5 SD) and has grown at 7 years to an OFC of 50.4 cm (11%). He developed seizures at 12 months of age which were well controlled with medications and subsequently weaned; he remained seizure free. The MRI was considered normal (not shown). He displayed motor and verbal delay with walking only beginning at 21 months, truncal hypotonia, and was not able to combine words at 23 months. There was a regression in the context of a febrile respiratory illness at 2-years with loss of words and walking. Over the next year he had mildly elevated creatine kinase levels just above normal levels, but he developed transaminitis with ALT levels varying between 2 and 15 times the upper limits of normal. He never developed an abnormal acyl carnitine profile. Over the next several years, the younger brother subsequently developed alternating hemiplegia. He required a G-tube for nutrition. His gait remained lordotic since age 3 years. His speech remained dysarthric with limited vocabulary and echolalia. He requires an individualized education program for school for both academics and behaviors. At 6 years his heart developed a right bundle branch block cardiac arrythmia.

The sister’s OFC at birth was 50% and for the first 6 months she was developing normally. By 8 months of age, she displayed severe constipation and central hypotonia. Her OFC reduced to the 12%. She developed epilepsy at 1 year of age which was intractable to medications. During this period, her development regressed with loss of sitting independently due to truncal ataxia. After 18 months, her seizures were controlled on monotherapy of valproic acid, and she became seizure free. G-tube was placed at 3 years for nutrition. At 4 years of age, she was in developmental preschool with a few single words, normal gross motor skills, but impaired fine motor skills. Acyl carnitine profiles and creatine kinase levels were consistently normal.

A recent review adroitly described *TANGO2* clinical manifestations of over 90 disease individuals (Schymick et al., 2022). The most common pathological variant is the deletion of exons 3 through 9 accounting for 42% of the pathological variants. Metabolic decompensation has been related to times of fasting or illness, like disorders of long-chain fatty acid oxidation but with ketosis in TANGO2 patients. The most common clinical features include episodes of metabolic crisis (87%), neurological abnormalities of dysarthria, ataxia, spasticity, hyper/hypotonia (87%), developmental delay (86%), intellectual disability (78%), rhabdomyolysis (75%), cardiac rhythm abnormalities (58%), hypothyroidism (57%), and epilepsy (51%). Most individuals developed an acute metabolic crisis prior to the age of 3 years (mean = 3.98 years).

Interestingly, the children in our case series with *TANGO2* variants all had epilepsy, developmental delays, and feeding difficulties with some form of metabolic instability without hypoketotic hypoglycemia. The patient in family 1 had the most common pathological variant in *TANGO2*, biallelic homozygous exon 3 through 9 deletions. She had an explosive onset of seizures and prolonged episodes of status epilepticus. Although epilepsy is common in this disease, the severity of her epilepsy has not been reported. She had liver involvement, hypothyroidism, and severe cognitive impairment. Unlike most *TANGO2* patients with the deletion of exons 3 through 9, there have not been episodes of rhabdomyolysis, heart arrhythmia, or hyperketotic hypoglycemia. Her MRI scans acutely demonstrated a diffuse pattern reminiscent of hypoxic-ischemic injury and abnormal lactate peak in the basal ganglia on MRS with subsequent diffuse atrophy like previous reports (Schymick, J. et al., 2022).

The three siblings from family 2 harbor biallelic heterozygous pathological variants in *TANGO2*, the most common variant of exon 3 through 9 deletion and a second unique variant c. 711-3G>G. The phenotype varied between the 3 children, with the index case having a severe epilepsy, epileptic spasms with hypsarrhythmia and subsequent medication resistant epilepsy. He also had a significant complex I enzymatic defect in the mitochondrial electron transport chain (Table 2). The other two siblings had variable epilepsy; the brother went into seizure remission while the girl has well controlled seizures on monotherapy. MRI in the index case of family 2 showed abnormal signal in the corticospinal tracts like a prior report (Berat et al., 2021).

Our cohort of patients did not express the findings associated with long-chain fatty acid oxidation defects typically seen in TANGO2 deficient patients. The detailed patient phenotypes demonstrate the wide variety of clinical findings, not only from previously described patients but within single family members having the same pathological variant.

## Discussion

In eukaryotes, organelle isolation enables specific and unique tasks. However, for proper whole cell physiological processing to occur, organelles must be able to communicate with each other. Our understanding of how this communication might occur has accelerated with developments of new technical advances in immunofluorescence with specific antibodies, electron microscopy with 3- dimension reconstruction, and cell fractionation techniques is beyond the scope of this study (reviewed in Giamogane et al., 2020). Proteins enmeshed and bridging opposing organelle membranes can act as communication conduits of product transfer from one compartment to another (Bernhard and Rouiller, 1956; Helle et al., 2013). Distinct structures have been identified for specific types of communication, which between the ER and mitochondria have been labeled MAM (Bernhard and Rouiller, 1956; Shore and Tata, 1977; Meier et al., 1981). Currently, several cellular MAMs involved in disease have been identified with specific alteration of mitochondrial functioning; calcium homeostasis, lipid transfer, mitochondrial dynamics, and mitophagy/apoptosis (Rizzuto et al., 1998; Youle and Narendra, 2011; Hoppins and Nunnari, 2012; Lev, 2012; Youle and van der Bliek, 2012; Wu et al., 2018). One more diverse pathway, *TANGO2*-related functioning has been associated with more diverse, but less clear cellular functioning (Bard et al., 2006).

We have identified patients with specific genetic variants in MAM proteins involved in calcium homeostasis, lipid transfer, mitochondrial dynamics, phospholipid remodeling and mitophagy/apoptosis and a protein involved with Golgi–ER communication with presumed mitochondrial communication.

Pathological genetic variants in *MICU1*, *PASC-2*, *SEARC1*, *CYP2U1*, and *TANGO2* are rare disorders and have been described as separate disease entities. Our patients had phenotypic and genetic similarities with the reported literature. The small numbers reported likely are an underestimation of the phenotypic variability, which can be seen in our described patients having identical genetic variants yet broader phenotype expression. The specific genetic variant findings were mostly previously unreported, but with the associated phenotype and computer modeling suggested deleterious change, are an indication of pathogenicity. All genes were noted by whole genome massive parallel sequencing and other possible candidates not reported. Combined with the phenotypes and laboratory testing and neuroimaging, the genetic variants for each patient are very likely pathological. The patients described in this report, all have neuroimaging abnormalities noting the importance of MAM in neurodevelopment.

Examining the patient phenotypes together, the patients’ histories start to unfold a set of common clinical findings. All our patients presented early in life with neurocognitive delay, developmental regression/stagnation, muscle problems, and varied involvement of other body systems. Biochemical findings are compatible with mitochondrial dysfunction. We think these findings suggest that there is a physiological interconnection between the MAMs described. Physiologically, there must be a logical communication between the various MAM functions. Just how this communication takes place is not currently known, but suggestive of interactions between MAMs. The breadth of phenotypes of our described patients would suggest that each MAM can produce a distinct disease phenotype, but also be involved in multisystem disorders that are due to the miscommunication of distinct functioning of a particular MAM. The proof of our hypothesis remains to be validated with more patients and better detection techniques.

## Conclusion

Specific disruptions in MAM functioning in calcium homeostasis, lipid transfer, mitochondrial dynamics of mitophagy, and apoptosis induce diseases that are beginning to highlight pivotal roles that rapid exchange of biological molecules maintains cellular health. This is a rapidly evolving field with new findings expanding what roles MAMs may play both in normal physiology and disease (Paillusson et al., 2016). The specific MAMs functions described here reveal the vast diversity of clinical presentations and disease progression between patients with the same disorder and between differing pathways.

As a group, all the individuals described have cognitive impairment, psychomotor delays, regression with stressor, and some degree of mitochondrial dysfunction. Although most individual did not have frank primary mitochondrial dysfunction, most do have findings suggestive of mitochondria involvement in their disease. The common findings within a particular MAM defect suggest possible isolated disease but the broad overlapping of phenotypes would be consistent with a much broader loss of interconnected functioning of MAMs.

## Data Availability

The authors acknowledge that the data presented in this study must be deposited and made publicly available in an acceptable repository, prior to publication. Frontiers cannot accept an article that does not adhere to our open data policies.
